# A Web Tool to Estimate Baseline Anti-Spike Monoclonal Antibody Efficacy Based on Regional Genomic Surveillance

**DOI:** 10.3390/v15051048

**Published:** 2023-04-25

**Authors:** Daniele Focosi

**Affiliations:** North-Western Tuscany Blood Bank, Pisa University Hospital, 56124 Pisa, Italy; daniele.focosi@gmail.com

**Keywords:** SARS-CoV-2, COVID-19, CoV-Spectrum, GISAID, anti-spike monoclonal antibodies, Evusheld

## Abstract

Drug appropriateness is a pillar of modern evidence-based medicine, but the turnaround times of genomic sequencing are not compatible with the urgent need to deliver treatments against microorganisms. Massive worldwide genomic surveillance has created an unprecedented landscape for exploiting viral sequencing for therapeutic purposes. When it comes to therapeutic antiviral antibodies, using IC_50_ against specific polymorphisms of the target antigen can be calculated in vitro, and a list of mutations leading to drug resistance (immune escape) can be compiled. The author encountered this type of knowledge (available from the Stanford University Coronavirus Antiviral Resistance Database,) in a publicly accessible repository of SARS-CoV-2 sequences. The author used a custom function of the CoV-Spectrum.org web portal to deliver up-to-date, regional prevalence estimates of baseline efficacy for each authorized anti-spike mAb across all co-circulating SARS-CoV-2 sublineages at a given time point. This publicly accessible tool can inform therapeutic choices that would otherwise be blind.

## 1. Introduction

The COVID-19 pandemic represented an unprecedented opportunity for targeted therapeutics, i.e., so-called directly acting antivirals (DAAs). DAAs are prone to resistance, which, in the case of antiviral monoclonal antibodies used for either pre-exposure prophylaxis or treatment [1], is referred to as “immune escape” [2].

Due to the high mutation rate of SARS-CoV-2, baseline resistance to clinically authorized anti-spike monoclonal antibodies (mAb) has been a moving target during the pandemic, making therapeutic choices often poorly informed. Unfortunately, the costs and turnaround times of current viral genome-sequencing platforms prevent the universal tailor-made assessment of baseline efficacy. Blind therapeutic choices are associated not just with a waste of money and an exposure to avoidable side effects, but also with a delay in appropriate treatment, which is a threat to patient health and comes with extra cost (prolonged hospitalization, additional testing, and treatment).

The delay between viral evolution and the corrective actions of drug regulatory authorities (such as the FDA or EMA), combined with a lack of repeat randomized clinical trials when new variants emerge, has led many to rely on in vitro data. The FDA has always trusted such in vitro data to promptly deauthorize treatments as soon as baseline resistance approached 50% of cases. That said, in large countries, nationwide decisions risk being unfair when different areas of the country are experiencing different dominant viral lineages with different baseline sensitivities. This is increasingly relevant given the ongoing co-circulation of several different viral lineages (so-called “variant soup” [3]).

Genomic surveillance has peaked at unprecedented levels for SARS-CoV-2, with more than 15 million sequences deposited in GISAID.org as of January 2023. This knowledge, which came at a high cost to society, can be exploited to inform therapeutic choices. Here, the author presents a web solution to assess baseline mAb efficacy in a given region by merging two publicly accessible databases, namely the Stanford University Coronavirus Antiviral Resistance Database (https://covdb.stanford.edu/, accessed on 1 March 2023) and the GISAID [4]-fed CoV-Spectrum.org web portal.

## 2. Materials and Methods

### 2.1. Identification of Clinically Authorized Anti-Spike Monoclonal Antibodies

The list of clinically authorized anti-spike mAb was extracted from web portals of the European Medicine Agency (EMA) (https://www.ema.europa.eu/en/human-regulatory/overview/public-health-threats/coronavirus-disease-covid-19/treatments-vaccines/covid-19-treatments, accessed on 1 March 2023) and the US Food and Drug Administration (FDA) (https://www.fda.gov/emergency-preparedness-and-response/mcm-legal-regulatory-and-policy-framework/emergency-use-authorization#coviddrugs, accessed on 1 March 2023).

### 2.2. Inferring Baseline Resistance to Anti-Spike Monoclonal Antibodies

The Stanford University Coronavirus Antiviral & Resistance Database is a comprehensively curated published database containing data about the susceptibility of SARS-CoV-2 variants to monoclonal antibodies and the plasma from previously infected and vaccinated people. It also records the spike mutations that have been selected by monoclonal antibodies and that have emerged in people experiencing prolonged infection [5]. It is publicly accessible at https://covdb.stanford.edu/, accessed on 1 March 2023. For each authorized anti-spike mAb, the author manually scanned mutations associated with a greater than five-fold increase in IC_50_ from the primary research listed in the search results provided at https://covdb.stanford.edu/search-drdb/?form_only, accessed on 1 March 2023. The cutoff was arbitrarily chosen by the author based on the existing literature. When primary research provided discordant results, the mutation was included only if the majority of primary studies provided an IC_50_ increase greater than five-fold.

### 2.3. Calculating the Prevalence of a Given Mutation in the Database

CoV-Spectrum.org is a web portal developed by the Computational Evolution (C-EVO) group at ETH Zurich [6]. In brief, it imports SARS-CoV-2 sequences deposited in GISAID, providing an advanced graphical and user-friendly interface that allows users to build their queries (“Collections”) at https://cov-spectrum.org/collections/add, accessed on 1 March 2023. The advanced query was built using rules published at https://cov-spectrum.org/about, accessed on 1 March 2023. For mAb cocktails, resistance was defined as the combined presence of at least one mutation conferring resistance to each of the ingredients. The same function is available at open.cov-spectrum.org, with source data from GenBank; the author relied on CoV-spectrum.org because GISAID includes more SARS-CoV-2 sequences than GenBank.

## 3. Results

Table 1 lists the spike mutations associated with greater than a five-fold reduction in IC_50_ for each of the selected anti-spike mAbs. Collection 75 is publicly available at https://cov-spectrum.org/collections/75, accessed on 1 March 2023. The output consists of the proportion of resistance lineages of all samples deposited in a given area (as reported in the sequence metadata) over a given time period for each mAb in the mAb cocktail. The lineages that are represented among the resistance pool are listed with the relative share according to PANGOLIN or NextStrain phylogenies. The entire list of mutations is managed as a single viral lineage, for which the growth advantage in time (with a confidence interval) can be calculated and eventually compared to a reference (“baseline”) lineage.

**Figure 1:**
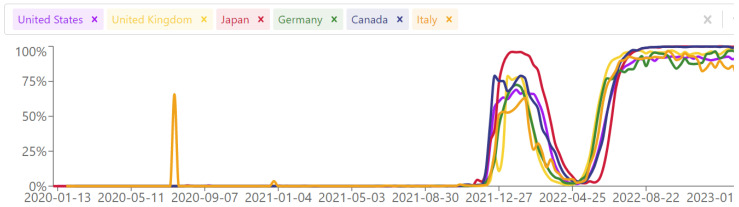
Prevalence of baseline resistance to Evusheld™ across different countries during the COVID-19 pandemic.

**Figure 2:**
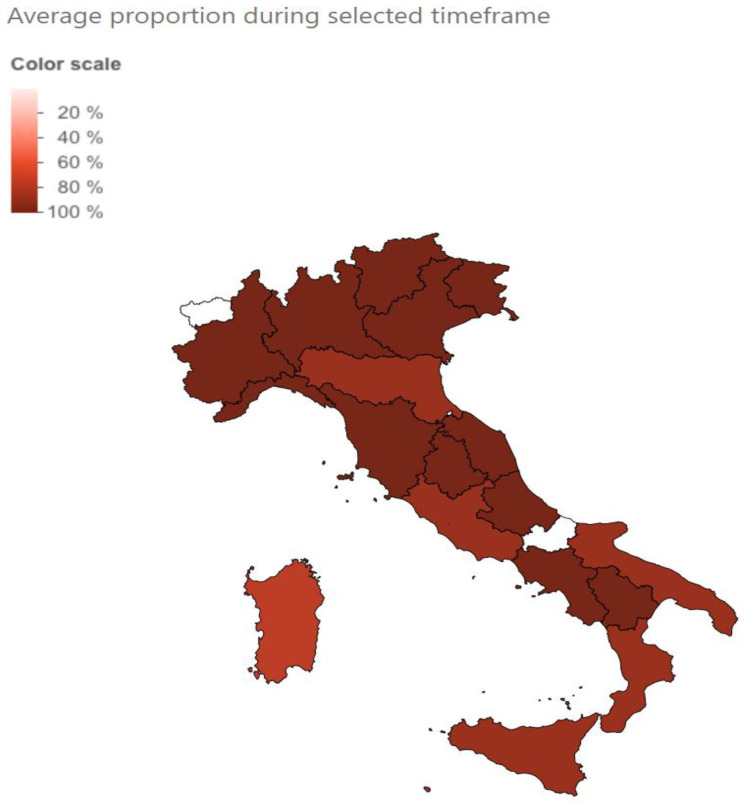
Prevalence of baseline resistance to Evusheld™ across regions of Italy in the 6 months from June to December 2022.

The web interface allows the user to further adjust the query, to filter by time period, by country, or by continent. Within the results for each country, maps of the regions are provided. An example of Evusheld™ resistance across different countries (Figure 1) and within Italian regions (Figure 2) is provided in Figure 1.

## 4. Discussion

Despite FDA deauthorization, many US physicians have continued the prescription of deauthorized mAbs [7], mostly based on personal beliefs in discrepancies between in vitro and in vivo activities. The latter phenomenon has been amplified across Europe [8], where, at time of publication, the EMA has never withdrawn an authorized mAb, and only sporadically issued alerts about possible resistance [9].

The present approach has several limitations. First, the author focused on viral neutralization to infer the therapeutic activity of anti-spike mAbs. This approach has been adopted by the FDA, and neutralizing antibody titers are, at time of publication, the best correlate of vaccine efficacy. In addition, a plethora of uncontrolled or poorly controlled literature advocates the continued efficacy of anti-spike mAbs based on marginal gains in surrogate endpoints [10]. Although binding affinity seems to be a robust surrogate endpoint, advocates of residual in vivo activity argue that the concentrations achieved by mAbs in vivo can overcome several degrees of baseline resistance to neutralization, and that alternative effector functions persist [11,12,13]. Neither arguments seem robust: with IC_50_ above 1000 [14], mAb binding is compromised, and effector functions other than neutralization invariably move from antigen binding.

Second, there is typically a delay of several days between patient sampling and sequence depositing in GISAID, which varies by country. It ranges from 9 days for Denmark to 52 for Japan, and is about 20 days for most of Europe and the USA (https://public.tableau.com/app/profile/raj.rajnarayanan/viz/MedianDaystoDepositSequences/Dashboard1, accessed on 1 March 2023). An extra 3–4 days delay should be added for data importing from GISAID into CoV-Spectrum. The cumulative delay seems to be negligible when considering the current pace at which regulatory authorities are adjusting recommendations; the example of Evusheld™ (deauthorized in the USA since January 2023 but still authorized in the EU, even though resistance has peaked at 100% since June 2022) is clear cut in terms of how a tool can largely anticipate the moves.

In conclusion, the author has shown how cross-database integration can inform the choices of prescribing clinicians, potentially minimizing inappropriate treatment and the associated costs and side effects while preserving regional differences.

## Figures and Tables

**Table 1:** Query structure for Collection 75.

Name	Query	Number of Sequences as of 31 January 2023	Submitted in the Past 10 Days
Cilgavimab resistance (ALL LINEAGES)	[1-of: S:346I, S:444E, S:444Q, S:444R, S:445A, S:446S, S:450D, S:450K, S:452R]	1.662.667	85.075
Tixagevimab resistance (ALL LINEAGES)	[1-of: S:371F, S:484A, S:484D, S:484K, S:486S, S:486V, S:493R, S:498R, S:990A, S:1009I]	1.757.503	90.423
Bebtelovimab resistance (ALL LINEAGES)	[1-of: S:444N, S:444Q, S:444T, S:445A, S:445F, S:445G, S:446D, S:446R, S:446V, S:499H, S:499R, S:449S]	365.241	40.544
Sotrovimab resistance (ALL LINEAGES)	[1-of: S:337H, S:337L, S:337R, S:337T, S:340A, S:340D, S:340G, S:340K, S:340Q, S:340V, S:356T, S:371F, S:371L, S:377K]	1.689.826	86.695
Casirivimab resistance (ALL LINEAGES)	[1-of: S:371F, S:405N, S:406D, S:406W, S:417E, S:417R, S:417N, S:417T, S:417V, S:445T, S:453F, S:455F, S:475R, S:484A, S:484K, S:484Q, S:486K, S:486L, S:486R, S:486S, S:486V, S:487R, S:493E, S:493K, S:493Q]	1.761.550	91.282
Imdevimab resistance (ALL LINEAGES)	[1-of: S:371F, S:371L, S:373P, S:406W, S:439K, S:440K, S:444L, S:444M, S:444N, S:444Q, S:444T, S:445A, S:446S, S:446V, S:450D, S:498H, S:498R, S:499S]	1.758.700	90.426
Casirivimab + imdevimab resistance (ALL LINEAGES)	[1-of: S:371F, S:405N, S:406D, S:406W, S:417E, S:417R, S:417N, S:417T, S:417V, S:445T, S:453F, S:455F, S:475R, S:484A, S:484K, S:484Q, S:486K, S:486L, S:486R, S:486S, S:486V, S:487R, S:493E, S:493K, S:493Q] and [1-of: S:371F, S:371L, S:373P, S:406W, S:439K, S:440K, S:444L, S:444M, S:444N, S:444Q, S:444T, S:445A, S:446S, S:446V, S:450D, S:498H, S:498R, S:499S]	1.758.195	90.390
Evusheld™ (tixagevimab + cilgavimab) resistance (ALL LINEAGES)	[1-of: S:371F, S:484A, S:484D, S:484K, S:486S, S:486V, S:493R, S:498R, S:990A, S:1009I] and [1-of: S:346I, S:444E, S:444Q, S:444R, S:445A, S:446S, S:450D, S:450K, S:452R]	1.661.161	84.579
bamlanivimab resistance (ALL LINEAGES)	[1-of: S:452R, S:484A, S:484D, S:484K, S:484Q, S:486R, S:486V, S:490L, S:490S, S:493H, S:493K, S:493L, S:493R, S:494P, S:494R]	1.712.794	89.253
etesevimab resistance (ALL LINEAGES)	[1-of: S:371F, S:371L, S:417N, S:417T, S:420N, S:456A, S:456K, S:456R, S:460K, S:460S, S:460T, S:460Y, S:475R, S:475V, S:486R, S:486L, S:486V, S:487R, S:493K, S:493L, S:493R]	1.758.046	90.373
bamlanivimab + etesevimab resistance	[1-of: S:371F, S:371L, S:417N, S:417T, S:420N, S:456A, S:456K, S:456R, S:460K, S:460S, S:460T, S:460Y, S:475R, S:475V, S:486R, S:486L, S:486V, S:487R, S:493K, S:493L, S:493R] and [1-of: S:452R, S:484A, S:484D, S:484K, S:484Q, S:486R, S:486V, S:490L, S:490S, S:493H, S:493K, S:493L, S:493R, S:494P, S:494R]	1.711.633	88.723
regdanvimab resistance	[1-of: S:452R, S:484K, S:493R]	1.548.951	72.218

## Data Availability

This manuscript generated now new dataset.
